# How Does Instability Affect Bench Press Performance? Acute Effect Analysis with Different Loads in Trained and Untrained Populations

**DOI:** 10.3390/sports11030067

**Published:** 2023-03-13

**Authors:** Moisés Marquina, Jorge Lorenzo-Calvo, Carlos García-Sánchez, Alfonso de la Rubia, Jesús Rivilla-García, Amelia Ferro-Sánchez

**Affiliations:** Sport & Training Research Group, Sports Department, Facultad de Ciencias de la Actividad Física y del Deporte (INEF), Universidad Politécnica de Madrid, 28040 Madrid, Spain

**Keywords:** resistance training, instability, balance, speed execution, core

## Abstract

(I) The execution of different sports involves a significant number of throws, jumps, or direction changes, so the body must be as stable as possible while performing a specific action. However, there is no classification of unstable devices and their influence on performance variables. Furthermore, the effect on athletes’ experience using instability is unknown. (II) The aim of this study was to analyze the power and speed parameters in bench press with different loads and unstable executions: (1) stable (SB), (2) with asymmetric load (AB), (3) with unstable load (UB), (4) on fitball (FB) and (5) on a Bosu^®^ (BB). A total of 30 male participants (15 trained and 15 untrained) were evaluated for mean propulsive speed (MPS), maximum speed (MS), and power (PW) with different types of external load: a low load (40% of 1RM), medium load (60% of 1RM), and high load (80% of 1RM) in each condition. Variables were measured with an inertial dynamometer. (III) The best data were evidenced with SB, followed by AB (3–12%), UB (4–11%), FB (7–19%), and BB (14–23%). There were no differences between groups and loads (*p* > 0.05) except in the case of MS with 60% 1RM, where trained participants obtained 4% better data (*p* < 0.05). (IV) Executions with implements and equipment such as fitball and Bosu^®^ do not seem to be the most recommended when the objective is to improve power or execution speed. However, situations where the load is unstable (AB and UB) seem to be a good alternative to improve stabilization work without high performance. Furthermore, experience does not seem to be a determining factor.

## 1. Introduction

Any sport is rarely played under stable conditions. The execution of different sports involves a significant number of throws, jumps or direction changes, so the body must be as stable as possible while performing a specific action. Therefore, training should aim to represent the requirements of the particular sport [1,2,3]. One of the approaches currently used in physical training, functional recovery and training in both team and individual sports is the use of unstable platforms, devices and unstable loads. Training in unstable conditions can provide a more effective transfer [4]. In recent years, training with unstable equipment has been shown to produce increased activation in trunk stabilizing muscles and is more beneficial for athletic performance and daily activities [5,6,7,8].

In the last decade, instability training has been included in athletes’ strength and conditioning programs and is constantly evolving and developing [9]. In fact, such has been its importance that its use and research have been extended to other populations, such as the military and astronauts [10]. However, the scientific community has focused on the study of muscle activations with destabilizing or suspension devices [11,12,13,14,15,16,17,18,19], and the effects of training in these situations on power and speed are unknown. 

Power is a strength manifestation that most athletes in different disciplines consider to be of greater importance for the performance of particular movements [20]. Power, besides being work in unit time, is force multiplied by speed, so it depends on two factors such as force and speed. Although it is an important component in athlete training, it is an equally important component in an exercise program for amateur athletes or recreational users of clubs or gyms [21]. Every activity performed, whether in sports or during everyday activities, requires individuals to react and generate force quickly to certain demands placed on our structure. Individuals must be trained at speeds that are functionally applicable in daily life and sport, reducing the risk of injury and improving performance, i.e., the ability to develop physical capacities to the maximum extent possible. In this way, instability training can help improve this functionality and adaptability in tasks that are not performed in a stable environment [20,21,22,23,24,25]. 

In high-performance sports, the asymmetry between body parts is evidenced by the repeated use of both active and passive movement devices. The specific requirements and movements of each sport involve a greater effort and development for one side of the body, highlighting the differences between the strong side and the skillful side. This asymmetry increases the differences between the two sides of the athlete’s body [26]. Excess asymmetry in muscle mass and strength between each side of the athlete’s body is associated with an increased risk of injury and also reduces the athlete’s motor potential [27,28,29]. Therefore, training with unstable environments, compensating for this decompensation provided by sport, may be beneficial.

So far, analysis of these situations has not established a differentiation in the experience of participants using untrained subjects in these types of situations [30]. There has only been one previous research study that has analyzed these differences [31]. Following the research line, this paper will try to shed some more light on this discipline. However, some studies have shown significant performance improvements in trained subjects. These studies emphasized the performance of strength exercises in unstable environments [32], in this case with exercises for the lower limb. The upper limb has also been analyzed, but without distinguishing between the experiences of the participants [32].

Bench press exercise is among the most commonly used exercises for upper body strength, hypertrophy, and power gains among recreational, endurance, and powerlifters [33]. Several studies have examined bench press exercises under different conditions and with different variables [34,35,36,37].

According to the literature reviewed, and based on the systematic review conducted [38], there is a lack of information, and studies, comparing the different stability conditions and their effect on performance variations. Only one study has compared and established differences between different conditions depending on the experience of the participants [31]. Furthermore, there is no scale or progression of the different instability situations, which determine which devices are more unstable depending on the bench press task. Similarly, there could be differences with different types of load. This paper attempts to provide an answer to these problems. Therefore, it seems necessary to investigate acute responses to training under unstable conditions based on performance variables such as power and speed. This acute effect will provide information on changes or adjustments produced during and immediately after a stimulus for applicability in training. The aim of this study was to analyze the differences in power and speed in the bench press with different unstable conditions in both trained and untrained male subjects; to determine whether experience in this task is relevant and whether there are differences between the different tasks; and to rank them from more stable to less stable depending on where the instability is located. In addition, to analyze whether differences are dependent on the load used.

## 2. Materials and Methods

### 2.1. Experimental Approach to the Problem

**Figure 1:**
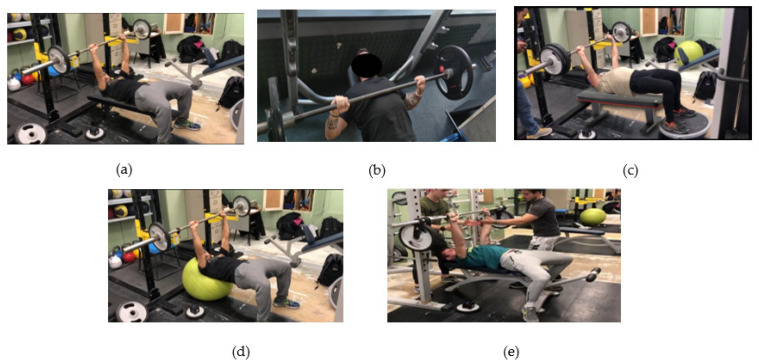
(**a**) Stable bench press; (**b**) asymmetric load; (**c**) lower instability on Bosu^®^; (**d**) upper instability on fitball; (**e**) unstable load.

**Figure 2:**
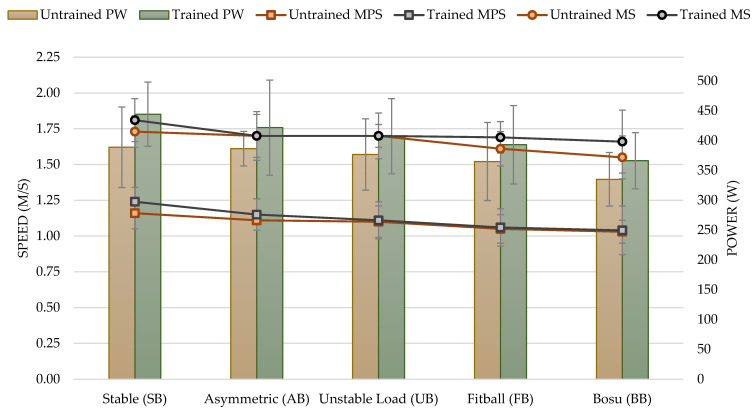
Mean propulsive speed (MPS), maximum speed (MS) measured in meters per second (m/s), and power (PW) measured in watts in the bench press exercise for untrained and trained groups based on experience with unstable performance tasks with 40% of 1RM. Notes: The figure shows the two variables analyzed in each execution. On the left *Y*-axis, the values of speed, and on the right *Y*-axis, the values of power. The colors show the different groups: trained in grey and untrained in red.

**Figure 3:**
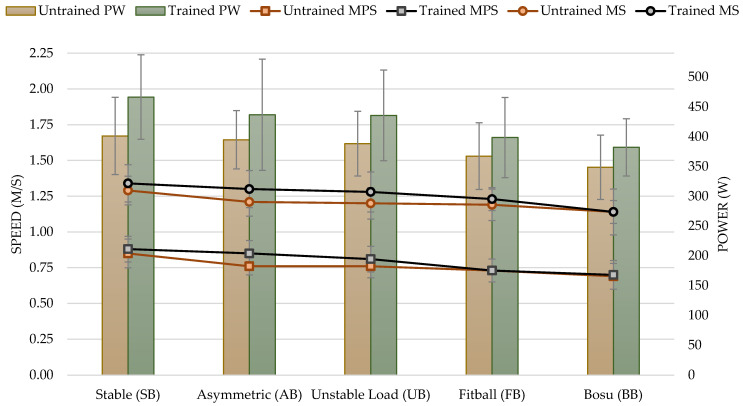
Mean propulsive speed (MPS), maximum speed (MS) measured in meters per second (m/s), and power (PW) measured in watts in the bench press exercise for untrained and trained groups based on experience for unstable performance tasks with 60% of 1RM. Notes: The figure shows the two variables analyzed in each execution. On the left *Y*-axis, the values of speed, and on the right *Y*-axis, the values of power. The colors show the different groups: trained in grey and untrained in red.

**Figure 4:**
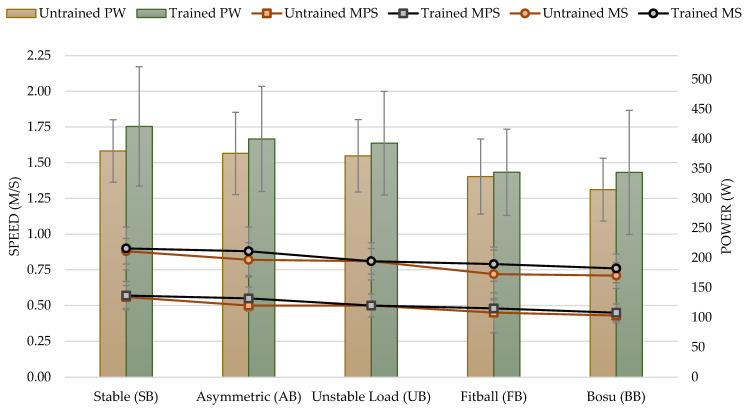
Mean propulsive speed (MPS), maximum speed (MS) measured in meters per second (m/s), and power (PW) measured in watts in the bench press exercise for untrained and trained groups based on experience for unstable performance tasks with 80% of 1RM. Notes: The figure shows the two variables analyzed in each execution. On the left *Y*-axis, the values of speed, and on the right *Y*-axis, the values of power. The colors show the different groups: trained in grey and untrained in red.

For this quasi-experimental research design, with an ad hoc protocol, an inter-subject comparison was used in 5 different conditions. The ad hoc protocol is a procedure that is needed and required at a specific time, for specific research needs, and tailor-made. The performance conditions with instability were: (1) stable bench-press condition (SB; Figure 1a), (2) bench press with asymmetric load (AB; Figure 1b), (3) bench press with unstable load (UB; Figure 1c), (4) bench press with back on fitball (SwissBall, Theragear, Basel, Switzerland) (FB; Figure 1d), and (5) bench press with feet on Bosu (BOSU^®^ Official Global Headquarters, Ashland, OH, USA) (BB; Figure 1d). The subjects performed the bench press exercise by evaluating the mean propulsive speed and power with 3 different loads for each of the unstable situations: light (40% of 1RM), medium (60% of 1RM), and high (80% of 1RM) [39]. Each task was performed with relative loads in a familiarization session in order to determine each of their individualized force–velocity profiles and the optimal percentage of work for the research. One of the most commonly investigated applications of velocity-based strength training is the ability to use movement velocity to determine the percentage of 1RM that is being lifted [40]. General equations of the load–velocity relationship that allow an estimation of % 1RM based on the velocity recorded at submaximal load were originally proposed for the bench press exercise [41]. Additionally, an incremental load test was performed to determine the 1RM of each participant to determine the external load for each percentage of load in each subject [42,43].

### 2.2. Participants/Sample

The sample size was calculated by calculating the statistical power (G*power 3.1.9.2) on a mean effect size of 0.25, using a 2 × 5 repeated measures design [44,45]. Corresponding to an α-level of 0.05 and the desired power (1-β) of 0.80 at the group level, the required sample size was 22 participants. To account for the drop out, we recruited 30 young adults. Enrolment for the research was completely voluntary. The 30 male participants were divided into 2 groups based on their previous experience with unstable training. In order to participate in the research, each of the participants had to meet the following criteria: (1) continuous strength training experience for a minimum of 3 years; (2) familiarity with the bench press exercise and frequently included it in their training; (3) no current or recent injury that prevented their physical activity for at least 6 months prior to the research; (4) no vigorous physical activity for at least 24 h prior to the research. The dividing factor between the groups was the experience in the use of instability training among the participants. The division into groups was based on previous experience in the use of unstable devices for strength training. Table 1 shows the descriptive data of the participants.

Membership in the trained or untrained group was determined by instability experience. The trained group of athletes had to demonstrate a minimum of 6 months of experience with unstable equipment and devices. These 6 months of instability training did not include tasks aimed at core improvement, proprioception, nor the use of these materials for rehabilitation or recovery from any type of injury. The reference of these 6 months of experience had to be continuous or with a time-lapse of less than 3 weeks. The instability experience criteria was not endorsed by any previous research, as the data between trained and untrained subjects have not been studied in depth. Therefore, this criteria was established based on the experience of the authors. Furthermore, professional (*n* = 2), elite (*n* = 2), or high-performance athletes (*n* = 3) were part of this group. These athletes competed in team sports (handball *n* = 3, track and field *n* = 1) and individual sports (paddle *n* = 2, powerlifting *n* = 1). All possible risks and benefits were explained and written informed consent was obtained prior to data collection. This study followed the Declaration of Helsinki (2013). All participants gave written informed consent after being informed of the possible risks. The research protocol and the consent forms were approved by the Ethics Committee of the Universidad Politécnica de Madrid (2020-062). Furthermore, the research was registered on ClinicalTrials.gov. (NCT04771494) (accessed on 22 February 2021).

### 2.3. Procedures

Participants completed a familiarization session and an incremental load test. Participants were asked to refrain from exercise for 24 h before each test. Participants completed a familiarization session with the different exercises two weeks prior to the experimental protocol, supervised by the researchers. A technical demonstration of movement was performed for each of the conditions. For familiarization, each participant had to perform 2 sets of 3 repetitions in each of the 5 study conditions. Breaks were 2 min after each set and exercise. The tasks were performed in a randomized order.

Each subject was instructed to perform the bench press movement using a standard 20 kg steel Olympic barbell (2.8 cm diameter, length 1.92 m) under 5 different conditions (see Figure 1): back on the bench, feet on the floor (stable); back on the bench, feet on the floor with asymmetric load on the bar (2 kg higher on the dominant side of the subject); the back on the bench, feet on the floor, with unstable loads (using discs attached with resistance bands); back on fitball (SwissBall, Theragear, Basel, Switzerland) feet on the floor (upper instability); back on the bench, feet on Bosu platform (BOSU^®^ Official Global Headquarters, Ashland, OH, USA) (lower instability).

In stable condition, each subject was placed supine on the bench with knees bent 90° and feet parallel on the floor, ensuring correct scapular retraction in the back, with gluteus and core contracted and feet flat on the floor, favoring curvature in the lumbar region (see Figure 1a). During lower body instability, subjects were instructed to maintain a bridge position with their shoulders resting on the bench. The subject’s feet were placed on the side of the Bosu^®^ platform with the knees bent at 90°. During all bridging postures, subjects were reminded to raise their pelvic girdle to be approximately parallel to their shoulders (see Figure 1e). Upper body instability was achieved by asking each subject to perform a bridge using a fitball that supports the shoulders through the first eight thoracic vertebrae. For this exercise, the subjects’ knees were bent at 90° with their feet on the floor (see Figure 1d).

#### 2.3.1. Incremental Load Test

After a specific warm-up, which included 5 min of cycling, followed by 5 min of mobility and dynamic flexibility exercises, and ending with approach series, the participants performed an incremental test up to 1RM in each of the selected tasks: bench press stable, asymmetric, with unstable loads, back on fitball, and feet on Bosu^®^. During the incremental test, the variables MPS, MS, and ROM were measured simultaneously with the device in each of the repetitions performed. 

A similar protocol was used for the incremental test as described in other studies [42,43]. Loads between 40% and 90% of the 1RM were used. The initial percentage ranged between 25% and 40% of the 1RM, depending on the subjects. The load increments depended on each of the exercises to be performed and the MPS of the previous set based on whether it was greater or less than 0.5 m/s^−1^. For lighter loads (MPS > 1.0 m/s^−1^), three attempts were made in each load, with a two-minute rest; two repetitions for medium loads (0.65 m/s^−1^ ≤ MPS ≤ 1.0 m/s^−1^) with a three-minute rest; and only one repetition for heavier loads (MPS < 0.65 m/s^−1^) with a five-minute rest. Spotters were present on both sides of the bar when high loads were lifted to ensure safety. These spotters help athletes, but do not excessively interfere; they observe, supervise, and support the athlete’s movement, only intervening when the athlete is unable to carry the weight.

For the stable and asymmetric bench press, starting with 40 kg on the bar, the increases were 20 kg if MPS > 0.65 m/s^−1^, between 5–10 kg if MPS between 0.45–0.65 m/s^−1^, and between 1–5 kg if MPS < 0.45 m-s^−1^. In the case of tasks performed with unstable loads, fitball, and Bosu^®^, starting with the bar’s weight, the increments were 20 kg if MPS > 0.65 m/s^−1^, 10 kg if MPS between 0.45–0.65 m/s ^−1^, and 5 kg if MPS < 0.45 m/s^−1^, using ballast.

#### 2.3.2. Experimental Protocol

For the experimental protocol, 3 repetitions were executed for the light load (40% RM; MPS > 1.0 m/s), resting for 1 min; 2 repetitions for the medium load (60% RM; 0.65 m/s ≤ MPS ≤ 1.0 m/s) resting for 2 min; and 1 repetition for the high load (80% RM; MPS < 0.65 m/s) with a 4 min rest [46]. In order to synchronize the execution times and movement cadence of both the concentric and eccentric phases, a metronome was used [47]. The initial position of the tasks was with the elbows fully extended, with a grip width slightly wider than the shoulder width of each participant. The execution continued with a lowering of the bar to chest height (over the nipples) and then a push to full elbow extension with a timing cadence of 2-1-0. This sequence determines 2 s of execution for the eccentric phase, a stop of 1 s when the bar is at chest height (for more reproducible and consistent measurements and thus minimizing the contribution of the rebound effect), and in the execution of the concentric phase it was to be performed at the highest possible speed, until the bar returned to the initial position.

#### 2.3.3. Data Extraction

Power (PW), maximum speed (MS), and Mean Propulsive Speed (MPS) were measured with an inertial dynamometer (Model TF-100; Ergotech T-Force System, Murcia, Spain) used and validated [48], with a calibration constant K = 0.4899 placed on the floor parallel to the Olympic bar, on which the end of the encoder cable was placed, before disc placement. To evaluate performance and program the strength training load according to execution speed, it is necessary to consider the “propulsive” phase in the concentric action. The propulsive phase determines the movement’s concentric velocity during which the acceleration evidenced by the load being moved is greater than the acceleration due to gravity (−9.81 m/s^2^) [41]. However, at high loads (>80% 1RM), a braking phase during the concentric phase is usually absent, so mean velocity (MV) is also a useful variable as is the mean propulsive velocity (MPV) as there are no substantial differences. Thus, both maximum, mean propulsive and mean velocity variables are equally reliable, but different contexts have to be taken into account. Therefore, when comparing subjects with different performances with the same absolute load (kg), the mean propulsive velocity “equalizes” the potentials, as it does not take into account the phase in which no force is produced (braking phase of the load), and does not average the velocity value over the entire performance, i.e., the propulsion phase plus the braking phase.

The encoder registers the displacement changes during the execution and therefore allows the speed to be calculated. To measure the power, measurement is made as a function of the speed and the changes in speed (acceleration). Thus, the hardware can measure the distance and the time to travel that distance. This enables the software to calculate speed and power for tasks with a completely vertical movement, without any horizontal components.

### 2.4. Statistical Analysis

Data analysis was performed with SPSS for Windows version 26 (IBM Corp., Armonk, NY, USA). The representation for quantitative variables is the mean (M) and standard deviation (SD). The Shapiro–Wilk (S-W) test was used to test for normality and Levene’s test for variance homogeneity; in addition, Mauchly’s test was performed to test for sphericity. Box’s test was performed to check the covariance matrix. If any variable did not meet the sphericity criteria, the Greenhouse–Geisser test was used. A two-factor (2 × 5) within-subject ANOVA analysis was used to test the effect of instability in the bench press. The Bonferroni test was applied for multiple a posteriori comparisons between the different groups. As an index of effect size, η^2^ [49] was used. The interpretation of η^2^ was classified as small for effect sizes >0.01 to <0.06, medium for >0.06 to <0.14 and large for >0.14 [50]. The significance level for all procedures was set at 0.05.

## 3. Results

### 3.1. Low Loads (40% of 1RM)

For the performance variables analyzed, there was a significant effect with a low load (40% of 1RM) based on instability. MPS (F_4,112_ = 12.84; *p* < 0.001; η^2^ = 0.314), MS (F_3,74_ = 7.01; *p* = 0.001; η^2^ = 0.200), and PW (F_4,112_ = 13.91; *p* < 0.001; η^2^ = 0.332). 

No significant differences were found for experience, which established that there was no difference between the trained and untrained groups: MPS (F_1,28_ = 1.2; *p* = 0.283), MS (F_1,28_ = 2.02; *p* = 0.166), and PW (F_1,28_ = 4; *p* = 0.055). 

There was also no difference in the interaction between MPS and participant experience: MPS (F_4,112_ = 0.62; *p* = 0.652), MS (F_3,74_ = 2.02; *p* = 0.125), PW (F_4,112_ = 1.33; *p* = 0.262).

At 40% load, the MPS reached with SB was significantly higher than with AB (*p* < 0.05), with UB, with FB, and with BB (*p* < 0.01 in all conditions). Furthermore, with AB, the MPS was significantly higher than with BB (*p* < 0.05). For the rest of the conditions, no significant differences were found (*p* > 0.05) (see Table 2). The results evidenced with AB, UB, FB, and BB showed a decrease of 5.83%, 7.50%, 12.50%, and 14.17%, respectively, compared to SB. 

MS achieved with SB was significantly higher than MS achieved with FB and BB (*p* < 0.01 in both comparisons). For the rest of the conditions, no significant differences were found (*p* > 0.05) (see Table 2). The results evidenced with AB, UB, FB, and BB showed a decrease of 3.95%, 3.95%, 6.78%, and 9.04%, respectively, compared to SB. 

The PW exerted with SB was higher than that achieved with FB (*p* < 0.05) and BB (*p* < 0.001). Furthermore, the power watts attained with AB were significantly higher than with BB (*p* < 0.001). Additionally, PW in UB was significantly higher than in BB (*p* < 0.001). For all other conditions, no significant differences were found (*p* > 0.05) (see Table 2). The results evidenced with AB, UB, FB, and BB showed a decrease of 3.88%, 4.15%, 8.75%, and 15.58%, respectively, when compared to SB (see Figure 2).

### 3.2. Medium Loads (60% of 1RM)

For the performance variables analyzed, there was a significant effect with a medium load (60% of 1RM) based on instability: MPS (F_4,112_ = 22.40; *p*< 0.001; η^2^ = 0.444), MS (F_4,112_ = 10.38; *p* = 0.001; η^2^ = 0.270), and PW (F_4,112_ = 24.40; *p* < 0.001; η^2^ = 0.466). 

Significant differences were also found according to experience in MS (F_1,28_ = 4.49; *p* = 0.043). The MS reached in the trained participants was significantly higher than in the untrained participants, with a difference of 4.29%. No differences were found according to the experience in MPS (F_1,28_ = 2.7; *p* = 0.113), neither PW (F_1,28_ = 4.17; *p* = 0.051).

There was no difference in the interaction between MPS and the experience of the participants: MPS (F_4,112_ = 2.12; *p* = 0.083), MS (F_4,112_ = 1.19; *p* = 0.318), and PW (F_4,112_ = 1.50; *p* = 0.206).

At 60% load, the MPS achieved with SB was significantly higher than that achieved with UB, with FB and with BB (*p* < 0.01 in all conditions). Furthermore, AB MPS was significantly higher than FB and BB (*p* < 0.01). Likewise, UB performance was significantly better than BB (*p* < 0.001). For the rest of the conditions, no significant differences were found (*p* > 0.05) (see Table 3). The results evidenced with AB, UB, FB, and BB showed a decrease of 8.05%, 9.20%, 16.09%, and 20.69%, respectively, compared to SB.

MS was significantly higher with SB than with FB (*p* < 0.05) and with BB (*p* < 0.01). Moreover, the AB execution was significantly better than BB (*p* < 0.05). Furthermore, UB performance was significantly better than BB (*p* < 0.01). For the rest of the conditions, no significant differences were found (*p* > 0.05) (see Table 3). The results evidenced with AB, UB, FB, and BB showed a decrease of 4.58%, 5.34%, 6.87%, and 12.98%, respectively, in comparison to SB.

The PW obtained for the 60% load with SB was significantly higher than the PW obtained with UB (*p* < 0.05), with FB, and with BB (*p* < 0.001 in both conditions). Likewise, AB power was significantly higher than FB and BB (*p* < 0.001). Furthermore, UB execution was significantly better than FB and BB (*p* < 0.001 in both conditions). For the rest of the conditions, no significant differences were found (*p* > 0.05) (see Table 3). The results evidenced with AB, UB, FB, and BB showed a decrease of 12.25%, 13.05%, 19.17%, and 22.87%, respectively, as compared to SB (see Figure 3).

### 3.3. High Loads (80% of 1RM)

For the performance variables analyzed, there was a significant effect with a high load (80% of 1RM) based on instability: MPS (F_3,91_ = 10.53; *p* < 0.001; η^2^ = 0.273), MS (F_3,84_ = 7.16; *p* < 0.001; η^2^ = 0.204), and PW (F_4,112_ = 13.80; *p* 0.001; η^2^ = 0.330).

No significant differences were found for experience, which established that there was no difference between the trained and untrained groups: MPS (F_1,28_ = 0.71; *p* = 0.406), MS (F_1,28_ = 1.52; *p* = 0.228), and PW (F_1,28_ = 1.07; *p* = 0.310).

No difference was found in the interaction between instability and participants’ experience, either: MPS (F_3,91_ = 1.42; *p* = 0.241), MS (F_3,84_ = 1.30; *p* = 0.279), and PW (F_4,112_ = 0.54; *p* = 0.704).

In performance at 80% load, the MPS achieved with SB was significantly higher than the one reached with UB, with FB (*p* < 0.01 in both conditions) and with BB (*p* < 0.001). Similarly, AB’s MPS was significantly higher than BB (*p* < 0.001). For the rest of the conditions, no significant differences were found (*p* > 0.05) (see Table 4). The results evidenced with AB, UB, FB, and BB showed a decrease of 8.77%, 14.04%, 15.79%, and 22.81%, respectively, compared to SB. 

MS achieved with SB was significantly higher than with FB and BB (*p* < 0.01 in both conditions). Furthermore, AB was significantly higher than FB (*p* < 0.01) and BB (*p* < 0.05). For the rest of the conditions, no significant differences were found (*p* > 0.05) (see Table 4). The results evidenced with AB, UB, FB, and BB showed a decrease of 3.37%, 8.99%, 15.73%, and 16.85%, respectively, in comparison to SB. 

PW achieved with SB was significantly higher than with FB and BB (*p* < 0.001 in both conditions). Likewise, AB was significantly higher than FB (*p* < 0.01) and BB (*p* < 0.001). Furthermore, UB was significantly superior to FB (*p* < 0.01) and BB (*p* < 0.001). For the rest of the conditions, no significant differences were found (*p* > 0.05) (see Table 4). The results evidenced with AB, UB, FB, and BB showed a decrease of 3.15%, 4.55%, 15.03%, and 17.72%, respectively, as compared to SB (see Figure 4).

## 4. Discussion

The purpose of this study was to analyze differences in power and speed in the bench press with different unstable conditions in trained and untrained male subjects. This is the first research to compare the strength and power performance of five bench press exercises commonly used in both the performance and health fields.

The most interesting finding of the present study was that there were no differences between the groups, except in the case of MS. Thus, it appears that experience with instability in the bench press may not be a determinant of performance variables. The main differences were between the stable and asymmetric conditions, compared to the fitball and Bosu^®^ performances. There was a progressive decrease in power and speed from the stable condition, followed by the asymmetric, unstable load, fitball, and Bosu^®^ executions. It should be noted that there were no significant differences between the stable condition and the asymmetric execution. Thus, it seems that asymmetric execution can be a good alternative and complement traditional bench press without a significant loss of performance and with a higher stabilization requirement.

The speed and power observed in the present research showed a decrease in these variables based on instability. These results support previous research which has shown that instability training does not improve the performance in power or movement speed [51,52]. Results in previous research with the push-up exercise showed differences between 13% and 38% as instability increased [31]. The differences in the current research are less than those in Marquina Nieto et al. [31], varying from 3–5% with the lowest instability (AB) to 17–22% with higher instability (BB and FB). Push-ups are an exercise with a similar movement pattern to the bench press, but the characteristics of both tasks are different. This decrease in power and speed may be due to the increased joint stiffness required to stabilize the joints involved in the bench press exercise. This does not occur in the case of push-ups. In addition, the high levels of external stability [53], which bench press involves, can be decisive. This external stability is only possible when training in a stable environment, such as the floor or a bench, and seems to be necessary to improve power performance. The differences between two similar exercises such as the push-up and the bench press may be since the barbell in the bench press has a greater instability. Instability generated by the external load of the exercise itself. This external load requires more coordination, so it already implies a more unstable environment than the execution of the push-up. In this way, it may decrease the effect on the increase in different unstable conditions [36].

However, not all the research that has studied the effectiveness of instability reported force reductions under unstable conditions [54,55]. In the present research, decreases in power and speed were observed, especially with the inclusion of materials such as fitball or Bosu^®^. However, there were no differences between AB and UB. The differences with these tasks compared to the stable condition were around 3–5%. In both cases, these data could be due to the fact that the initial position and the supports were performed on a stable bench and with the feet on the ground, as with the stable condition. However, the load location varies with respect to the SB. These results suggest that the supports to exert the force are more relevant than the instability of the load. In turn, with both AB and UB, they have shown improvements in muscle activation compared to the stable condition [54,55]. In terms of muscle activation, AB reduced chest and shoulder muscle activity on the less loaded side, while maintaining muscle activity on the loaded side [56]. With UB, a similar EMG amplitude was observed in the shoulder, but a greater pectoral and external oblique activity compared to the traditional bench press [57]. Therefore, these results show that with a minimal decrease in performance with AB it can be a good alternative for training. In addition, some training methods such as offset, could be a good alternative and complement traditional training. The offset training method is based on performing resistance exercises with an asymmetrical external load position. Unlike unilateral exercises with contralateral or ipsilateral external load placement, the offset training method assumes a bilateral but asymmetrical external load position. The greater the external load placed on one side of the body, the greater the athlete’s postural control, as well as lateral and rotational stability requirements [37].

Differences in the conditions were found with equipment such as fitball and Bosu^®^, which are similar to those found in other studies. The results are shown in other research that examined the fitball bench press and reported a very similar percentage decrease in terms of mean power (10.3% [52]; 9.9% [54]; and 12.9% [51]). In this study, the power decrease with the three types of load ranged from 9% at 40% load to 19% loss, with the highest loss reported at 60% of 1RM. At 80% of 1RM, it was 15%. Our data, therefore, seem to be in line with those previously demonstrated. The possible reason for this high loss when using these devices may be because participants must stabilize their upper body on an unstable surface to provide firm support for the contracted muscles. This additional task may compromise the muscle twitch that acts on the bar. In the stretch-shortening cycle, concentric muscle action does not immediately succeed eccentric muscle action, the stored energy is dissipated and lost as heat and the stretch-reinforcement reflex is also not activated. Around this inflection point, where the eccentric phase becomes concentric, the maximum strength is produced. Their less intense contraction not only prolongs the change of direction in the movement but also, due to the lower maximal strength, impairs the accumulation in elastic energy. The consequence is lower speed and power in the subsequent concentric phase [58]. On the other hand, already in an intervention study, results showed significant improvements in mean power during chest presses on a Swiss ball at weights up to 60.7% 1RM after 4 and 8 weeks of instability resistance training [59].

The differences in MPS, MS, and PW were notable when performing the bench press on a Bosu^®^, varying between 16% and 23%. Therefore, it could be suggested that instability located in the lower part of the body in the bench press exercise produces greater losses in performance than if the instability is located in the upper part of the body. These data are consistent with previous findings [31]. However, in this case, the differences were much wider than in the present study and with different task such as push-ups. However, there are not enough studies that have analyzed the bench press with lower body instability.

In terms of speed decrease, the differences were greater with the MPS, with all loads. For MS, the differences were lower, even very small, between the stable condition and the execution with asymmetric load and unstable load. However, there is barely any evidence for execution speed. Koshida et al. [54], showed a loss of 9%. This is the only study that has analyzed the execution speed with the bench press. Our data show a higher decrease, in line with the push-up exercise [31]. These losses could be due to the findings of Adkin et al. [60], as fear of falling can lead to a reduction in running speed. Thus, muscle stabilization compromises gains in strength, power, and execution speed [61]. It should also be noted that new movement patterns are learnt at low speed, whereas sport-specific motor actions are executed at high speed [62]. It should be noted that as the external load increased, especially with the execution of high loads, the safety of the task decreased and, on many occasions, the participants had to rebalance several times before performing the lift. Higher loads also lead to greater instability regardless of the situation or condition. However, it was found that the fitball generated a kind of rebound, a kind of stretch-shortening cycle, which also threw them off balance between repetitions.

Undoubtedly, adaptations in strength, power, and speed are determined by the intensity of the resistance established [63]. However, with the loads analyzed, there does not appear to be a clear difference in the load where the greatest differences between performances were found was at 60% of 1RM. The loads of 40% and 80% of 1RM showed smaller differences between the conditions. Differences in execution speed at load between 60% and 80% are noticeable in the case of fitball execution. These differences increase considerably at 80% load (15–20%) while at 60% load, they were smaller (8–15%).

It was interesting to find no differences between the different groups in any of the situations and variables analyzed, which in the bench press exercise leads us to believe that previous experience is not a determining factor. However, it is noteworthy that at a low load (40%), the data from the trained participants showed a greater loss of performance as the instability increased, which did not affect the untrained participants as much. Furthermore, at all loads, the power values showed a greater loss of performance as the instability increased compared to the untrained participants. These results are not in line with a previous study, in which differences were found in all conditions between the trained and untrained groups [31]. This may be due to the difference in the tasks, although they have the same movement pattern, the bench press task is more unstable than the push-up and this could be a reducing factor in the experience of the participants.

The findings of the present study were in line with previous studies in which no differences were observed between training groups with different stability requirements [30,64,65]. Sparkes and Behm [66] demonstrated similar improvements in the 3-RM bench press on a stable (bench) or unstable (Swiss ball) surface after training on a stable training program (training machines) or an unstable training program (free weights or unstable surfaces). However, in these cases, no differentiation was made between trained and untrained subjects. They did differ from what was found in the push-up exercise, where the differences according to the experience of the participants were clear [31].

It should be noted that the use of high loads (80%) in the most unstable conditions (fitball and Bosu^®^) required the participation of two assistants and involved, at times, several attempts to achieve balance before starting the lifts. Therefore, it may not be advisable to use it without adequate supervision and assistance.

Certain limitations and strengths of the study must be recognized. The external structure of the movement (i.e., kinematics and movement torque) was not investigated. In addition, the activity of the agonist, antagonist, and core muscles was not assessed. Only one familiarization session was performed, and there is not enough information on the short- or long-term effects of performing asymmetric lifts. The participants had an average resistance training experience of approximately 10 years and excellent test-retest reliability was observed for all conditions. Furthermore, only men with a high level of resistance training were recruited and the results are not necessarily generalizable to other populations. Other populations (e.g., women, adolescents, the elderly or athletes) may experience strength imbalances between limbs and evidence different results.

## 5. Conclusions

Execution with implements and equipment such as fitball and Bosu^®^ does not seem to be the most advisable when the objective is to improve power or execution speed. In addition, these devices may not be safe when higher loads are used and may not be effective. However, situations where the load is unstable, seem to be a good alternative to improve stabilization work without a highly decreasing performance. Furthermore, experience does not seem to be a determining factor as there is no differentiation between participants trained in instability and those not trained. Finally, instability located in the lower extremities seems to compromise bench press performance to a greater extent.

## 6. Practical Applications

So far, no objective method has been described to quantify the intensity of exercise on unstable surfaces. This could be a useful tool to monitor exercise intensity in unstable conditions. In addition, a classification of the most unstable tasks was established.

Evidence-based findings may be beneficial in balancing strength levels and avoiding a continuous emphasis on the use of the stronger side. Additionally, slight instability can be beneficial for strengthening weak links or even for rehabilitation.

In addition, situations such as asymmetric loads can help activities that require asymmetric strength development (e.g., alpine skiing or martial arts, team sports, or activities of daily living), and complement their traditional resistance training with asymmetric or unstable loads. Therefore, a type of training such as offset training could be very interesting for the development of physical capacities. 

On the other hand, the use of equipment such as fitball or Bosu^®^ is not recommended when the objective is to improve speed or power.

## Figures and Tables

**Table 1:** Sample descriptive data.

Group	Age (Years)	Body Mass (kg)	Body Height (cm)
Untrained (*n* = 15)	26.73 ± 4.31	77.80 ± 4.44	176.40 ± 2.80
Trained (*n* = 15)	27.54 ± 3.57	81.49 ± 9.67	179.58 ± 8.95

**Table 2:** Descriptive statistics for mean propulsive speed (MPS), Maximum Speed (MS) measured in meters per second (m/s), and power (PW) measured in watts (W) based on instability at light loads (40%).

	Untrained MPS						Trained MPS							All Participants		
	N	M	SD	% Dif	IC—95%		N	M	SD	% Dif	IC—95%		% Dif. Groups	M	SD	% Dif.
					LL	UP					LL	UP				
Stable (SB)	15	1.16	0.11		1.11	1.22	15	1.24	0.10		1.18	1.30	6.45%	1.20	0.11	
Asymmetric (AB)	15	1.11	0.07	4.31%	1.06	1.16	15	1.15	0.11	7.26%	1.10	1.20	3.48%	1.13 ^a^	0.10	5.83%
Unstable Load (UB)	15	1.10	0.11	5.17%	1.04	1.17	15	1.11	0.13	10.48%	1.05	1.18	0.90%	1.11 ^a^	0.12	7.50%
Fitball (FB)	15	1.05	0.10	9.48%	0.99	1.11	15	1.06	0.13	14.52%	1.00	1.12	0.94%	1.05 ^a^	0.11	12.50%
Bosu (BB)	15	1.03	0.08	11.21%	0.96	1.10	15	1.04	0.17	16.13%	1.00	1.11	0.96%	1.03 ^ab^	0.11	14.17%
	**Untrained MS**						**Trained MS**							**All Participants**		
	**N**	**M**	**SD**	**% Dif**	**IC—95%**		**N**	**M**	**SD**	**% Dif**	**IC—95%**		**% Dif. Groups**	**M**	**SD**	**% Dif.**
					**LL**	**UP**					**LL**	**UP**				
Stable (SB)	15	1.73	0.11		1.67	1.84	15	1.81	0.15		1.74	1.89	4.42%	1.77	0.14	
Asymmetric (AB)	15	1.70	0.15	1.73%	1.66	1.80	15	1.70	0.17	6.08%	1.61	1.78	0.00%	1.70	0.16	3.95%
Unstable Load (UB)	15	1.70	0.08	1.73%	1.63	1.77	15	1.70	0.16	6.08%	1.63	1.77	0.00%	1.70	0.13	3.95%
Fitball (FB)	15	1.61	0.12	6.94%	1.55	1.67	15	1.69	0.11	6.63%	1.63	1.75	4.73%	1.65 ^a^	0.12	6.78%
Bosu (BB)	15	1.55	0.15	10.40%	1.46	1.65	15	1.66	0.22	8.29%	1.57	1.76	6.63%	1.61 ^a^	0.19	9.04%
	**Untrained PW**						**Trained PW**							**All Participants**		
	**N**	**M**	**SD**	**% Dif**	**IC—95%**		**N**	**M**	**SD**	**% Dif**	**IC—95%**		**% Dif. Groups**	**M**	**SD**	**% Dif.**
					**LL**	**UP**					**LL**	**UP**				
Stable (SB)	15	388.93	67.68		354.36	423.51	15	444.40	53.90		421.51	467.29	12.48%	415.50	51.69	
Asymmetric (AB)	15	386.60	28.92	0.60%	363.71	409.49	15	421.87	79.67	5.07%	384.59	459.15	8.36%	399.36	72.94	3.88%
Unstable Load (UB)	15	376.87	59.91	3.10%	339.59	414.15	15	407.60	62.96	8.28%	373.03	442.17	7.54%	398.27	64.93	4.15%
Fitball (FB)	15	365.07	65.56	6.13%	330.37	399.76	15	393.20	65.64	11.52%	358.51	427.90	7.15%	379.13 ^a^	66.03	8.75%
Bosu (BB)	15	335.13	45.04	13.83%	310.77	359.50	15	366.40	47.07	17.55%	342.04	390.76	8.53%	350.77 ^abc^	47.98	15.58%

Notes: a = Significant differences compared SB; b = Significant differences compared AB; c = Significant differences compared UB; MPS = mean propulsive speed; MS = maximum speed; PW = power; M = mean; SD = standard deviation; % Dif = percentage difference between conditions; IC—95% = Interval confidence—95%; LL = lower limit; UP = upper limit; % Dif. Groups = percentage difference between groups. SB = stable bench press; AB = asymmetric bench press; UB = unstable load bench press; FB = fitball bench press; BB = Bosu bench press.

**Table 3:** Descriptive statistics for Mean Propulsive Speed (MPS), Maximum Speed (MS) measured in meters per second (m/s), and Power (PW) measured in watts (W) based on instability at medium loads (60%).

	Untrained MPS						Trained MPS								All Participants		
	N	M	SD	% Dif	IC—95%		N	M		SD	% Dif	IC—95%		% Dif. Groups	M	SD	% Dif.
					LL	UP						LL	UP				
Stable (SB)	15	0.85	0.10		0.80	0.95	15	0.88		0.09		0.83	0.93	3.41%	0.87	0.09	
Asymmetric (AB)	15	0.76	0.06	10.59%	0.72	0.81	15	0.85		0.09	3.41%	0.81	0.89	10.59%	0.80	0.09	8.05%
Unstable Load (UB)	15	0.76	0.08	10.59%	0.72	0.80	15	0.81		0.09	7.95%	0.76	0.85	6.17%	0.79 ^a^	0.09	9.20%
Fitball (FB)	15	0.73	0.08	14.12%	0.69	0.77	15	0.73		0.08	17.05%	0.69	0.77	0.00%	0.73 ^ab^	0.08	16.09%
Bosu (BB)	15	0.69	0.09	18.82%	0.65	0.75	15	0.70		0.10	20.45%	0.64	0.74	1.43%	0.69 ^abd^	0.10	20.69%
	**Untrained MS**						**Trained MS**								**All Participants**		
	**N**	**M**	**SD**	**% Dif**	**IC—95%**		**N**	**M**		**SD**	**% Dif**	**IC—95%**		**% Dif. Groups**	**M**	**SD**	**% Dif.**
					**LL**	**UP**						**LL**	**UP**				
Stable (SB)	15	1.29	0.10		1.23	1.35	15	1.34		0.13		1.27	1.40	3.73%	1.31	0.12	
Asymmetric (AB)	15	1.21	0.10	6.20%	1.16	1.26	15	1.30		0.13	2.99%	1.24	1.36	6.92%	1.25	0.13	4.58%
Unstable Load (UB)	15	1.20	0.11	6.98%	1.13	1.26	15	1.28		0.14	4.48%	1.22	1.35	6.25%	1.24	0.13	5.34%
Fitball (FB)	15	1.19	0.11	7.75%	1.13	1.25	15	1.23		0.08	8.21%	1.19	1.28	3.25%	1.22 ^a^	0.09	6.87%
Bosu (BB)	15	1.14	0.08	11.63%	1.07	1.21	15	1.14		0.16	14.93%	1.07	1.21	0.00%	1.14 ^abc^	0.13	12.98%
	**Untrained PW**						**Trained PW**								**All Participants**		
	**N**	**M**	**SD**	**% Dif**	**IC—95%**		**N**	**M**		**SD**	**% Dif**	**IC—95%**		**% Dif. Groups**	**M**	**SD**	**% Dif.**
					**LL**	**UP**						**LL**	**UP**				
Stable (SB)	15	401.13	64.81		365.22	437.05	15	466.33		70.87		430.42	502.25	13.98%	473.73	74.51	
Asymmetric (AB)	15	394.73	48.95	1.60%	355.32	434.15	15	436.67		93.33	6.36%	397.25	476.08	9.60%	415.70	76.27	12.25%
Unstable Load (UB)	15	388.27	54.21	3.21%	353.35	423.19	15	435.60		76.02	6.59%	400.68	470.52	10.87%	411.93 ^a^	69.19	13.05%
Fitball (FB)	15	367.27	56.01	8.44%	334.52	400.01	15		398.53	67.32	14.54%	365.78	431.28	7.84%	382.90 ^abc^	62.89	19.17%
Bosu (BB)	15	348.67	54.10	13.08%	321.61	375.72	15		382.07	48.04	18.07%	355.00	409.12	8.74%	365.37 ^abc^	53.06	22.87%

Notes: a = Significant differences compared SB; b = Significant differences compared AB; c = Significant differences compared UB; d = Significant differences compared FB; MPS = mean propulsive speed; MS = maximum speed; PW = power; M = mean; SD = standard deviation; % Dif = percentage difference between conditions; IC—95% = Interval confidence—95%; LL = lower limit; UP = upper limit; % Dif. Groups = percentage difference between groups. SB = stable bench press; AB = asymmetric bench press; UB = unstable load bench press; FB = fitball bench press; BB = Bosu bench press.

**Table 4:** Descriptive statistics for mean propulsive speed (MPS), Maximum Speed (MS) measured in meters per second (m/s), and power (PW) measured in watts (W) based on instability at high loads (80%).

	Untrained MPS						Trained MPS							All Participants		
	N	M	SD	% Dif	IC—95%		N	M	SD	% Dif	IC—95%		% Dif. Groups	M	SD	% Dif.
					LL	UP					LL	UP				
Stable (SB)	15	0.56	0.08		0.51	0.61	15	0.57	0.10		0.53	0.63	1.75%	0.57	0.09	
Asymmetric (AB)	15	0.50	0.04	10.71%	0.46	0.53	15	0.55	0.08	3.51%	0.51	0.58	9.09%	0.52	0.07	8.77%
Unstable Load (UB)	15	0.50	0.08	10.71%	0.46	0.53	15	0.50	0.08	12.28%	0.46	0.53	0.00%	0.49 ^a^	0.07	14.04%
Fitball (FB)	15	0.45	0.14	19.64%	0.40	0.52	15	0.48	0.06	15.79%	0.44	0.52	6.25%	0.48 ^a^	0.11	15.79%
Bosu (BB)	15	0.43	0.05	23.21%	0.40	0.46	15	0.45	0.06	21.05%	0.42	0.48	4.44%	0.44 ^ab^	0.06	22.81%
	**Untrained MS**						**Trained MS**							**All Participants**		
	**N**	**M**	**SD**	**% Dif**	**IC—95%**		**N**	**M**	**SD**	**% Dif**	**IC—95%**		**% Dif. Groups**	**M**	**SD**	**% Dif.**
					**LL**	**UP**					**LL**	**UP**				
Stable (SB)	15	0.88	0.09		0.81	0.95	15	0.90	0.15		0.82	0.97	2.22%	0.89	0.13	
Asymmetric (AB)	15	0.82	0.12	6.82%	0.74	0.89	15	0.88	0.17	2.22%	0.81	0.95	6.82%	0.86	0.14	3.37%
Unstable Load (UB)	15	0.81	0.09	7.95%	0.75	0.87	15	0.81	0.13	10.00%	0.75	0.87	0.00%	0.81	0.11	8.99%
Fitball (FB)	15	0.72	0.17	18.18%	0.65	0.79	15	0.79	0.12	12.22%	0.73	0.84	8.86%	0.75 ^a^	0.11	15.73%
Bosu (BB)	15	0.71	0.09	19.32%	0.66	0.76	15	0.76	0.10	15.56%	0.69	0.83	6.58%	0.74 ^a^	0.14	16.85%
	**Untrained PW**						**Trained PW**							**All Participants**		
	**N**	**M**	**SD**	**% Dif**	**IC—95%**		**N**	**M**	**SD**	**% Dif**	**IC—95%**		**% Dif. Groups**	**M**	**SD**	**% Dif.**
					**LL**	**UP**					**LL**	**UP**				
Stable (SB)	15	379.73	52.59		1595.37	1790.55	15	421.07	100.27		1946.03	2145.08	9.82%	400.4	81.43	
Asymmetric (AB)	15	375.67	69.07	1.07%	1277.28	1470.51	15	399.93	88.33	5.02%	1772.64	1969.69	6.07%	387.8	78.88	3.15%
Unstable Load (UB)	15	371.60	60.78	2.14%	1252.20	1424.87	15	392.80	86.91	6.71%	1615.51	1791.59	5.40%	382.2	74.47	4.55%
Fitball (FB)	15	336.60	63.18	11.36%	1197.77	1340.87	15	344.00	72.43	18.30%	1503.87	1649.81	2.15%	340.2 ^abc^	84.79	15.03%
Bosu (BB)	15	314.87	52.82	17.08%	1104.57	1254.13	15	343.80	104.27	18.35%	1447.90	1600.42	8.41%	329.43 ^abc^	64.02	17.72%

Notes: a = Significant differences compared SB; b = Significant differences compared AB; c = Significant differences compared UB; MPS = mean propulsive speed; MS = maximum speed; PW = power; M = mean; SD = standard deviation; % Dif = percentage difference between conditions; IC—95% = Interval confidence—95%; LL= lower limit; UP= upper limit; % Dif. Groups = percentage difference between groups. SB = stable bench press; AB = asymmetric bench press; UB = unstable load bench press; FB = fitball bench press; BB = Bosu bench press.

## Data Availability

Not applicable.
